# Multilayer representation of collaboration networks with higher-order interactions

**DOI:** 10.1038/s41598-021-85133-5

**Published:** 2021-03-11

**Authors:** E. Vasilyeva, A. Kozlov, K. Alfaro-Bittner, D. Musatov, A. M. Raigorodskii, M. Perc, S. Boccaletti

**Affiliations:** 1 Moscow Institute of Physics and Technology, 9 Institutskiy Per., Dolgoprudny, 141701 Moscow, Russia; 2 P.N. Lebedev Physical Institute of the Russian Academy of Sciences, 53 Leninsky Prosp., 119991 Moscow, Russia; 3 Unmanned Systems Research Institute, Northwestern Polytechnical University, Xi’an, 710072 China; 4 Departamento de Física, Universidad Técnica Federico Santa María, Av. España 1680, Casilla 110V, Valparaíso, Chile; 5 Russian Academy of National Economy and Public Administration, Pr. Vernadskogo, 84, 119606 Moscow, Russia; 6 Caucasus Mathematical Center, Adyghe State University, ul. Pervomaiskaya, 208, 385000 Maykop, Russia; 7 Mechanics and Mathematics Faculty, Moscow State University, Leninskie Gory, 1, 119991 Moscow, Russia; 8 Institute of Mathematics and Computer Science, Buryat State University, ul. Ranzhurova, 5, 670000 Ulan-Ude, Russia; 9 Faculty of Natural Sciences and Mathematics, University of Maribor, Koroška Cesta 160, 2000 Maribor, Slovenia; 10 Department of Medical Research, China Medical University Hospital, China Medical University, Taichung, 404332 Taiwan; 11 Complexity Science Hub Vienna, Josefstädterstraße 39, 1080 Vienna, Austria; 12 CNR-Institute of Complex Systems, Via Madonna del Piano 10, 50019 Sesto Fiorentino, Italy; 13 Universidad Rey Juan Carlos, Calle Tulipán s/n, Móstoles, 28933 Madrid, Spain

**Keywords:** Complex networks, Statistical physics

## Abstract

Collaboration patterns offer important insights into how scientific breakthroughs and innovations emerge in small and large research groups. However, links in traditional networks account only for pairwise interactions, thus making the framework best suited for the description of two-person collaborations, but not for collaborations in larger groups. We therefore study higher-order scientific collaboration networks where a single link can connect more than two individuals, which is a natural description of collaborations entailing three or more people. We also consider different layers of these networks depending on the total number of collaborators, from one upwards. By doing so, we obtain novel microscopic insights into the representativeness of researchers within different teams and their links with others. In particular, we can follow the maturation process of the main topological features of collaboration networks, as we consider the sequence of graphs obtained by progressively merging collaborations from smaller to bigger sizes starting from the single-author ones. We also perform the same analysis by using publications instead of researchers as network nodes, obtaining qualitatively the same insights and thus confirming their robustness. We use data from the arXiv to obtain results specific to the fields of physics, mathematics, and computer science, as well as to the entire coverage of research fields in the database.

## Introduction

Scientific collaboration networks are an important subset of complex social networks^1–4^. They document patterns of collaboration that we have formed to do research, and to arrive at new scientific discoveries and breakthroughs that drive technological progress and innovation in our societies. The outstanding importance of science and progress for the wellbeing of modern human societies, together with the consistent definition of scientific collaboration that is accurately documented in published research^5^, has given rise to a rich plethora of research dedicated to the determination of structure and function of scientific collaboration networks^6–12^. Along the same lines, citation networks^13–15^, bipartite author-publication networks^16–19^, hypergraphs of scientific output^20^, as well as simplicial descriptions of publications and corresponding topological methods^21,22^, have also been considered and studied in much detail.

However, despite the fact that traditional complex networks have come a long way in improving our understanding of economic, infrastructural, technological, as well as social and computer networks^23–26^, the past decade has witnessed the rise of the narrative that the majority of these networks do not exist in isolation. Rather, many are coupled together and therefore should be best described as interdependent or multilayer networks^27,28^. Indeed, it has been shown that even tiny changes or a failure in one network layer can lead to a catastrophic cascade of much more significant failures across many other network layers^29^. It was a seminal discovery, and while some argued that processes in different network layers could simply be added up and described as a conglomerate process on a single-layer network, it soon became clear that, as is in general true for complex systems, the whole is not simply the sum of its parts^30–32^. Multilayer networks have since found applications for better understanding epidemic spreading^33,34^, vaccination^35^, evolution of cooperation^36^, and biological organization at different scales^37^.

**Figure 1:**
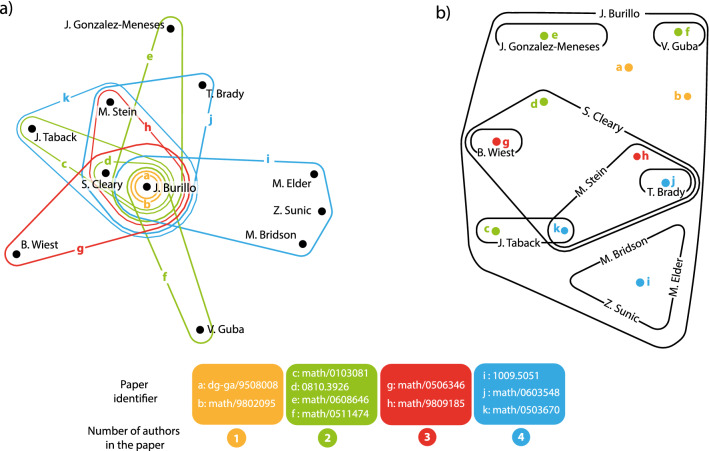
Schematic illustration of the co-authorship hypergraph **(a)** and of the dual hypergraph **(b)**. In panel **(a)** nodes are authors, and hyperlinks are co-authored Manuscript. The hyperlinks are labeled with letters and colours. The legend at the bottom of the Figure reports for each letter the corresponding Manuscript’s identifier in the ArXiv. In the legend, moreover, Manuscripts are grouped in coloured boxes, and different colours stand for a different number of coauthors: yellow papers are authored by a single Scholar, whereas green, red and blue Manuscripts are co-authored by two, three and four Scholars, respectively. Panel **(b)** contains a sketch of the dual representation, where nodes are now papers [labeled with the same colours and letters than in panel **(a)**], and links are labeled with the name of the authors who participated in the co-authorship of the Manuscripts.

We here map published scientific papers in a multilayer network: scientists are nodes in all layers, and a link between two nodes in the $\hbox {j}^{th}$ ($j>1$) layer stands for the participation of the corresponding two scientists in a publication jointly written by *j* co-Authors. This way, single-author publications form the first layer, two-author publications form the second layer, three-author publications form the third layer, and so on. In doing so, the layers themselves already hold important information about the collaboration. It is namely easy to argue that two researchers that are the only two authors on a publication have a much stronger link than two researchers that have co-written a paper that has several hundred authors, as is often the case in high-energy physics publications. Multilayer collaboration networks defined in this way thus naturally take into account the problems that are commonly associated with unweighted single-layer collaboration networks^12,38–41^. Moreover, if we aggregate all the layers, we simply obtain the complete scientific collaboration network, but with the added value that, as we coalesce the layers obtained with ever larger collaboration sizes, we obtain novel microscopic insights into the representativeness of researchers within different teams and their links with others, and we can follow the maturation of topological features and the relevance each particular layer has in this process.

Another important distinction of our research to traditional scientific collaboration networks is that we consider higher-order interactions to describe the networks. This is irrelevant for the first and second layer, but becomes theoretically much more convenient for the subsequent layers, where three or more coauthors are naturally connected by a single higher-order link—a hyperlink – rather than a series of 2nd-order links connecting pairs of researchers consecutively with one another. Although the value of higher-order interactions has been recognized already in the early 70s by Atkin^42,43^ and Berge^44^, the interest peaked only recently with mounting inability to converge on what constitutes a group or how to define it consistently in the realm of social network analysis^45–49^, and the interested reader can find a comprehensive account on the role of higher-order interactions in networked systems in Ref.^49^.

Here we use the formalisms of multilayer and higher-order networks, often also called hypergraphs, to study the maturation of different topological characteristics of collaboration networks in physics, mathematics, and computer science by using the arXiv database^50^. And we also consider the entire coverage of research fields in the same database. The question that we seek to answer is, how many layers does one need to obtain a proper and robust description of the collaboration network? Or equivalently, is it possible to describe the collaboration network by taking into account publications with only a couple of authors, for example up to layer four or five?

**Figure 2:**
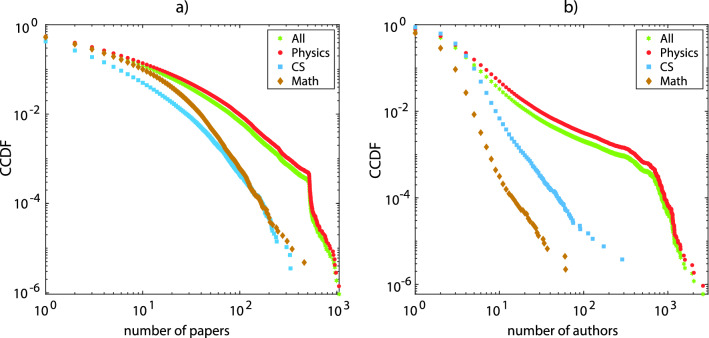
**(a)** Complementary cumulative distribution functions (CCDF, see text for definition) for the primal graphs obtained from the data-set. The distributions are functions of the nodes’ degree distributions for *H*($H_{phys},\ H_{math},\ H_{cs}$) and of hyperedges’ degree distributions for the respective dual hypergraphs. **(b)** CCDF for the dual graphs, which are functions of the hyperedges’ degree distribution in *H*($H_{phys},\ H_{math},\ H_{cs}$) and of the nodes degree distribution in the respective dual hypergraphs. Curves are coloured according to the different speciality from which papers are extracted from the data-set (see the colour code at the top right of each panel).

## Results

We refer to the information publicly available in the arXiv (https://arxiv.org/, https://github.com/mattbierbaum/arxiv-public-datasets/) database^50^. Data parsing was also made according to^50^. From the database, metadata on 1,679,779 articles were downloaded. Then, information about 1,068,043 unique authors was parsed.

Let *N* be the number of authors in the database. The main idea is to represent the data-set as a primal $H = (V, E_H)$ co-authorship hypergraph, in which $V=\{v_1,\dots ,v_N\}$ is the set of nodes (authors) and $E_H$ is a set of hyperedges accounting for articles. In this representation, an article co-authored by *d* authors corresponds then to an hyperedge grouping the *d* authors of the paper, as it is schematically depicted in Fig. 1a. In Fig. 1a nodes are therefore labeled with the name of the authors, whereas coloured hyperlinks are labeled by the corresponding paper identifier in the arXiv (with different colours, moreover, standing for different numbers of coauthors). Notice that this representation allows to distinguish the case of two (or a limited group of) researchers that are the only authors of a publication and therefore they supposedly have a strong ties, from that of two (or a limited group of) researchers that just participate in huge collaboration projects giving rise to papers that have several hundred authors.

Moreover, the primal hypergraph $H = (V, E_H)$ can be associated to a dual hypergraph $H^* = (V^*, E^*_H)$ in which $V^*$ is the set of articles and $E^*_H$ groups papers written by the same author (in collaboration with others, or individually), as schematically depicted in Fig. 1b. One can also introduce a kind of “pairwise approximation” of *H*, given by a undirected graph $G = (V,E_G)$ where an edge between authors reflects the existence of a joint paper (independently on the number of coauthors). Therefore, each hyperedge of *H* corresponds to a clique in *G*. With the same spirit, the dual graph $G^* = (V^*,E^*_G)$ is the pairwise approximation of $H^*$ where nodes correspond to articles and existence of an edge indicates that two articles have at least one joint author.

The hypergraph *H* (and its dual $H^*$) as well as the graphs *G* and $G^*$ can be viewed as multilayer networks with layering index defined by the number of article’s coauthors, and represented in Fig. 1 by different colours assigned to different papers (yellow denoting Manuscripts authored by a single scholar, green papers co-authored by two scholars, etc...). Then, one can operate a progressive fusion of such layers, and obtain the hypergraph (graph, dual hypergraph and dual graph respectively) *H*(*n*) (*G*(*n*), $H^*(n)$, $G^*(n)$), where only papers with no more than *n* coauthors are considered. Let ${\bar{n}}$ be the number of maximal layer in the statistics, and let us simplify the notations further by writing $H({\bar{n}})$ ($G({\bar{n}})$, $H^*({\bar{n}})$,$G^*({\bar{n}})$) as *H* (*G*,$H^*$,$G^*$). *H*, *G*, $H^*$ and $G^*$ are the “asymptotic” graphs and they are actually the “classical” representations given to collaborations’ data, where all level of co-authorship (as much those implying just a few scholars as those implying instead thousands of scholars) are mixed together, and whose main properties have been largely characterized by the definition and calculation of a wealth of topological measures.

**Figure 3:**
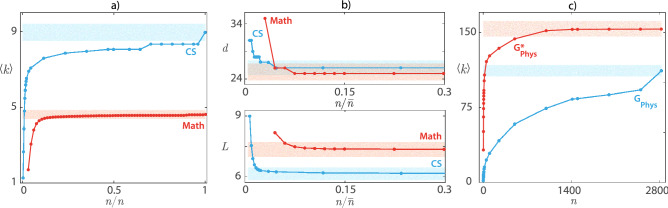
Illustration of the maturation process of different topological features. Panel **(a)**: the average degree $\langle k \rangle$ vs. the normalized fusion index $n/{\bar{n}}$ (see text for definitions), for the areas of mathematics (light red curve) and computer science (light blue curve). The horizontal light red and light blue bars stand for the (plus or minus) $\varepsilon = 0.05$ errors around the respective asymptotic values $\langle k \rangle ({\bar{n}})$. Panel **(b)**: the upper (lower) sub-panel reports the evolution of the diameter *d* (of the shortest path *L*) in the areas of mathematics (light red curve) and computer science (light blue curve). *d* maturates at layer 3 in the area of mathematics and at layer 10 in the area of computer science; *L* instead maturates at layer 4 in mathematics and again at layer 8 in computer science. Notice that different topological features maturate at different fusion stages. Panel **(c)**: the average degree $\langle k \rangle$ in the area of physics vs. the fusion index *n*, for the direct graph $G_{phys}$ (light blue line) and for the dual graph $G^*_{phys}$ (light red line).

Our idea is, instead, that such topological measures are actually *maturating* as one progressively fuse the distinct layers. In other words, we suggest that there exists a given ${\tilde{n}}$ at which each specific network’s topological property maturates, i.e. it assumes the asymptotic value which is calculated on *H*, *G*, $H^*$ and $G^*$. Obviously, such maturation level may be different for different fields of cooperation (as processes of scientific collaboration formation vary from field to field) and for different topological measures as well, and it is of great interest to study how distinct topological properties emerge at distinct levels of fusions (i.e. taking into account only proper subsets of the original data, where the number of coauthors of a given Manuscript is limited).

Finally, it has to be noticed that all articles in the database are related to eight main areas (physics, mathematics, quantitative biology, computer science, quantitative finance, statistics, electrical engineering and systems science, economics) and in the present study we give our new representation of co-authorship networks for the following fields (in parentheses we report the notation for each one of the obtained asymptotic graphs):

- physics ($H_{phys}$, $G_{phys}$ and the dual ones),
- math ($H_{math}$, $G_{math}$ and the dual ones),
- computer science ($H_{cs}$, $G_{cs}$ and the dual ones),
- all eight areas together (*H*, *G* and the dual ones).

### A first characterization of the hypergraphs

A first rough characterization of the primal and dual graphs is shown in Fig. 2, where we report the complementary cumulative distribution function (CCDF) for *H*($H_{phys},\ H_{math},\ H_{cs}$) in panel (a) and for *G*($G_{phys},\ G_{math},\ G_{cs}$) in panel (b).

The CCDF is defined with the following expression:

1 $$\begin{aligned} {\mathrm{CCDF}}(x)=1-F(x), \end{aligned}$$

where *F*(*x*) is the cumulative distribution function. If the tail of the distribution is fitting a the power-law, then

$$\begin{aligned} {\mathrm{CCDF}}(x)\sim x^{-(\gamma + 1)},\ x>x_m,\ \gamma >0 \end{aligned}$$

where $x_m$ is a proper parameter, and $\gamma$ can be estimated as the slope of the linear fit in a log–log scale. In Fig. 2a,b we report the CCDF for nodes’ and hyperedges’ degree distributions of *H*, $H_{phys},\ H_{math},\ H_{cs}$ and of their dual graphs. From the figures it is apparent that the different graphs deviate from a power law in their tails. The distributions in physics (red curves) can be seen as consisting of two different parts which actually seems to correspond to different power law exponents. Most likely, such a property is due to experimental works in huge collaborations. Hyperedges’ degree distributions in math and CS deviate from the power law only in tails. The distributions corresponding to the entire database display the same features as those in physics, as papers related to this science prevail in the arXiv collection.

The results shown in Fig. 2 point to the fact that there are papers with an extremely high number of coauthors. However, as already discussed in the Introduction, real patterns of authors’ interactions are unlikely to be determined by such huge collaborations. Therefore, it seems reasonable to analyse how papers with large numbers of authors affect the network properties, or equivalently to analyse the maturation properties of the multilayer networks defined in the previous sub-section.

### The maturation process of topological features in the multilayer graph

The main objective of our study is to compare stabilization and maturation patterns of co-authorship networks describing scientific cooperation in different fields. To this purpose, we analyze how different topological properties change when the layer index *n* changes.

Let *x*(*n*) be some property (i.e., some topological measure) of a graph *G*(*n*). To simplify the notations, we omit the argument for the case of the maximal layer ${\bar{n}}$, and we write $x = x({\bar{n}})$. We say that the specific property *x*(*n*) is maturated at the layer ${\tilde{n}}(x)$ if:

2 $$\begin{aligned} {\tilde{n}}(x) = \arg \min _n\left\{ n: \forall k\ge n \longrightarrow \frac{|x(k) - x|}{x}\le \varepsilon \right\} , \end{aligned}$$

where $\varepsilon$ is a small constant accounting for an acceptable accuracy (i.e., a tolerable difference). In all our calculations, we use $\varepsilon = 0.05$.

In order to illustrate the concept of *maturation*, Fig. 3 anticipates some of the major points and conclusions of our Manuscript, and reports three panels, each one displaying the maturation behavior (or the absence of maturation) of important topological features, as the fusion index *n* of layers increases.

Precisely, Fig. 3a compares the behavior of the average degree $\langle k \rangle$ versus $n/{\bar{n}}$ for the areas of mathematics (light red curve) and computer science (light blue curve). Normalization in the horizontal axis is needed because the two areas have actually distinct maximum numbers ${\bar{n}}$ of layers. It is clearly seen that $\langle k \rangle$ *maturates* rather early in the area of mathematics: $\langle k \rangle (n/{\bar{n}})$ is a monotonically increasing curve which attains its asymptotic value (the value at $n={\bar{n}}$) already at layer ${\tilde{n}}=8$. The horizontal light red bar in panel (a), indeed, stands for the (plus or minus) $\varepsilon = 0.05$ error around the asymptotic value $\langle k \rangle ({\bar{n}})$, and it is evident that the curve $\langle k \rangle (n/{\bar{n}})$ stays inside the error area for all values of ${\tilde{n}} \le n \le {\bar{n}}$. At variance, the average degree never maturates in the area of computer science, as witnessed by the light blue line in Fig. 3a: once again the horizontal light blue bar indicates the (plus or minus) $\varepsilon = 0.05$ error around the asymptotic value $\langle k \rangle ({\bar{n}})$, but now the curve $\langle k \rangle (n/{\bar{n}})$ never enters the error area before attaining its asymptotic value at $n={\bar{n}}$.

Different topological features may maturate at different values of ${\tilde{n}}$, as illustrated in panel (b) of Fig. 3. Namely, the upper (lower) part of panel (b) reports the evolution of the diameter *d* (of the shortest path *L*) in the areas of mathematics (red curve) and computer science (light blue curve). *d* maturates at layer 3 in the area of mathematics and at layer 10 in the area of computer science; *L* instead maturates at layer 4 in mathematics and again at layer 8 in computer science. It is seen, moreover, that different fusion stages at which maturation in different areas takes place is not the simple consequence of the normalization of the fusion index *n* to the relative maximum number of layers in the area.

Finally, panel (c) of Fig. 3 anticipates another important conclusion of our study: in some cases dual graphs, where hyperlinks connect publications instead of coauthors, may represent a better rendering of collaboration networks, in that some topological features maturate in dual graphs, whilst they never maturate in the direct graphs. This is illustrated with reference to the average degree $\langle k \rangle$ in the area of physics: it is clearly seen that the curve $\langle k \rangle ({n})$ for the direct graph $G_{phys}$ (light blue line) does not display any maturation feature, whereas $\langle k \rangle ({n})$ (light red line) maturates at layer 574 in $G^*_{phys}$.

It is essential to remark that the calculation of some graph’s topological measure for all layers *n* may have an associated very high computational demand. Therefore, the networks *G*(*n*) and $G^*(n)$ are here analysed using a rather sparse grid, and after that the dependencies are interpolated using splines. The procedure, however, do not affect (nor distorts) the conclusions which we are offering below.

### General properties

The natural starting point is the analysis of the networks’ global substructures.

Let *CC* be a set of network’s connected components, and *LCC* be the set of vertices in the largest connected component. The following notation can be introduced:

- $$m = |E_G|$$,
- $$N_{CC} = |CC|$$,
- $$s_{LCC} = \frac{|LCC|}{N}$$.

Table 1 reports the maximal number of layers (${\bar{n}}$), the number of nodes (*N*), the number of edges (*m*), the number of connected components ($N_{CC}$), the relative size of the largest connected component ($s_{LCC}$), and the maturation layer’s numbers for all these features (${\tilde{n}}(\cdot )$).

The first significant feature which should be noticed is the differentiation in ${\bar{n}}$ for the different disciplines. Namely, physics corresponds to the highest value of ${\bar{n}}$ (2831 layers). Moreover, the number of nodes *N* in physics maturates quite late if compared with math and CS, therefore a consistent number of Scholars in this field write papers only in rather big collaborations. In contrast, in math one has see the smallest number of layers (67), and not only *N*. Furthermore, not only *N* maturates early (already at level 5) in this field, but even the edges’ number maturates at level eight, which implies that focusing only on papers with no more than eight authors one has an almost complete description of the graph representing the math discipline. For the other graphs, one sees instead that the number of edges significantly changes at all levels of the fusion process, up to the final layers.

**Table 1:** Maturation indices and maturation values of the main general properties of primal and dual graphs.

		All	Math	CS	Phys
	${\bar{n}}$	2831	67	427	2831
*G*	$N,\ \times 10^6$	1.07	0.21	0.28	0.71
	${\tilde{n}}(N)$	26	5	9	44
	$m,\ \times 10^7$	4.11	0.05	0.13	3.96
	${\tilde{n}}(m)$	–	8	–	–
	$N_{CC},\ \times 10^4$	5.18	2.74	2.08	2.48
	${\tilde{n}}(N_{CC})$	11	4	6	23
	$s_{LCC}$	0.90	0.76	0.79	0.93
	${\tilde{n}}(s_{LCC})$	7	4	6	7
$G*$	$N,\ \times 10^6$	1.68	0.44	0.26	1.08
	${\tilde{n}}(N)$	8	4	6	10
	$m,\ \times 10^7$	10.11	0.82	0.59	8.27
	${\tilde{n}}(m)$	522	5	9	574
	$N_{CC},\ \times 10^4$	5.18	2.7	2.08	2.48
	${\tilde{n}}(N_{CC})$	11	4	6	23
	$s_{LCC}$	0.94	0.86	0.86	0.95
	${\tilde{n}}(s_{LCC})$	4	3	5	4

All notations and definitions are reported in the text. The symbol “–” reflects the fact that the property does not maturate, implying that significant changes in the property’s value occur at all fusion indices, up to the final layer (the reported values are therefore the “asymptotic” ones obtained by fusing all layers).

Another notable feature which appear from Table 1 is related to the number of connected components. This property maturates relatively early for all fields, as well as for the whole graph. Therefore, besides the largest connected component, the general backbone of the other part of the graph is formed by many clusters (connected components) each one containing a relatively small number of papers. On the other hand, the largest connected component consists of about 80% of nodes for the fields of math and CS and 93% of nodes in physics. The relative size of the LCC in the whole graph is 90%, which means that the LCC of the whole graph contains all authors from the LCC’s of the different fields’ graphs. This notion follows from the fact that if we suppose that the smallest LCC from Table 1 (the one of math) is not included into LCC of the whole graph than size of LCC of the whole graph should be not more than $(1.07 - 0.76\cdot 0.21)/1.07=0.85$. As a conclusion, there is an important role of interdisciplinary links connecting Authors from different fields. In Table 1 we also report the same properties for the dual graphs. The remarkable result is that even the edge number maturates for all fields in the dual graphs. Most likely this occurs because an extremely large number of authors is much more frequent than an extremely large number of papers written by a particular author, and moreover such papers have to have different sets of coauthors in order to contribute to the number of edges. Therefore, papers with large number of authors contribute large cliques in *G* but not in its dual graph, and therefore, in this context, the dual graph constitutes a better representation of the social collaborations than its primal counterpart.

### Degree distribution

The second step of our analysis is the description of the local networks’ properties, and we start with the study of the degree distributions. Let $k_i(n),\ i=1,\dots ,N(n)$ be the degree of node *i* in *G*(*n*). Our results show that, for all graphs analysed in the current study, the probability distribution functions (PDFs) of the degree *k* are fat-tailed, with tails well described by a power law scaling with exponent $\gamma$:

3 $$\begin{aligned} p(k) \sim \frac{1}{k^{\gamma }}, \end{aligned}$$

Table 2 reports the values of the mean degree in the four graphs studied ($\langle k \rangle$) and the estimated tail exponents $\gamma$ for the corresponding degree distributions. The only field in which we see a maturation of the mean degree is math, which is also characterized by the highest tail exponent. The fat-tailed nature of the degree distribution is the most likely reason for the absence of maturation in the mean degree, as well as in the tail exponent estimation. In the cases of the whole graph and of the graph of physics, one sees that maturation, however, occurs at a very high value of the fusion index. Even if such distribution maturates, sample estimations of such values are often very sensitive to additional observations or data. Table 2 reports also the results for the dual graphs. One immediately sees that the mean degrees of all graphs under consideration maturate and, moreover, the exponents of the respective power law distributions are significantly higher. Therefore, once again the dual graphs estimates seem to provide a more accurate characterization.

**Table 2:** Maturation indices and maturation values of the degree distribution’s properties for the primal and dual graphs.

		All	Math	CS	Phys
	${\bar{n}}$	2831	67	427	2831
*G*	$\langle k \rangle$	77.01	4.62	8.97	111.51
	${\tilde{n}}(\langle k \rangle )$	–	8	–	–
	$\gamma$	1.7	3.6	2.6	1.6
	${\tilde{n}}(\gamma )$	498	–	–	1411
$G*$	$\langle k \rangle$	120.54	36.65	44.33	153.54
	${\tilde{n}}(\langle k \rangle )$	522	5	8	574
	$\gamma$	2.8	3.3	3.9	2.6
	${\tilde{n}}(\gamma )$	756	2	7	495

All notations and definitions are reported in the text. The symbol “–” reflects the fact that the property does not maturate, implying that significant changes in the property’s value occur at all fusion indices, up to the final layer (the reported values are therefore the “asymptotic” ones obtained by fusing all layers).

### Network clustering

One of the most important graph’s properties is clustering. Such a measure, indeed, accounts for networks’ transitivity, and in the context of co-authorship graph it describes how often two coauthors of one particular author are coauthors themselves in other papers. Quantification of clustering’s effects can be obtained by measuring two different coefficients: the global and the local clustering ones. The global clustering coefficient is defined by the following expression:

4 $$\begin{aligned} C=\frac{3\#K_3}{\#P_2}, \end{aligned}$$

where $\#K_3$ is the number of triangles in the graph and $\#P_2$ is the number of connected chains of length two.

The local clustering coefficient of vertex *i* is instead calculated as

5 $$\begin{aligned} c_i = \frac{|\{j,k\in E_G: j,k\in N_i\}|}{C_{|N_i|}^2}, \end{aligned}$$

where $E_G$ is the set of edges of graph G, $N_i$ is the set of *i*’s neighbors. I.e. local clustering coefficient measures the fraction of connected triples around node *i*. The overall graph clustering property $\langle c \rangle$ can be obtained by averaging the local clustering coefficient of Eq. (5) over all nodes:

6 $$\begin{aligned} \langle c \rangle = \frac{1}{N} \sum _{i \in V}c_i. \end{aligned}$$

One can easily see that the expression  (Eq. (4)) can be rewritten as

7 $$\begin{aligned} C = \frac{\sum _{i\in V} C_{|N_i|}^2 c_i}{\sum _{i\in V}C_{|N_i|}^2}. \end{aligned}$$

From Eq. (7) it follows that in calculating the global clustering coefficient the higher is the degree of the nodes the higher its weight in the average, whereas $\langle c \rangle$ takes all nodes equivalently. Therefore, the higher the difference between *C* and $\langle c \rangle$ is, the higher is the non-uniformity of clustering distribution between nodes.

**Table 3:** Maturation indices and maturation values of the graphs’ clustering properties.

		All	Math	CS	Phys
	${\bar{n}}$	2831	67	427	2831
*G*	*C*	0.57	0.24	0.70	0.57
	${\tilde{n}}(C)$	–	–	–	–
	$\langle c \rangle$	0.65	0.48	0.69	0.68
	${\tilde{n}}(\langle c \rangle )$	10	5	6	15
$G*$	*C*	0.27	0.78	0.62	0.26
	${\tilde{n}}(C)$	546	4	7	522
	$\langle c \rangle$	0.72	0.76	0.68	0.70
	${\tilde{n}}(\langle c \rangle )$	5	2	3	7

All notations and definitions are reported in the text. The symbol “–” reflects the fact that the property does not maturate, implying that significant changes in the property’s value occur at all fusion indices, up to the final layer (the reported values are therefore the “asymptotic” ones obtained by fusing all layers).

Table 3 shows the clustering coefficients estimation and maturation for all primal and dual graphs. The first notable feature is that the global clustering coefficient never maturates, while the averages of the local clustering coefficient always do. This naturally follows from the fact that papers from the last layers are associated with larger numbers of additional triangles, and they also contribute a huge number of edges, thus enlarging nodes’ degrees significantly, which are then used to calculate weights in the average of the global clustering coefficient [see Eq. (7)]. The smallest values of the clustering coefficients are in the field of math, which also can be distinguished for significant difference between *C* and $\langle c \rangle$. Namely, in math global clustering is two times less than the averaged local one. Therefore in maths nodes with high degree are less clustered then the ones with small degree.

In dual graphs, both global and local clustering coefficients maturate. Moreover, the averaged local clustering coefficients maturate earlier than the ones calculated in the primal graphs. Furthermore, the levels of maturation in the whole graph, in physics and in maths are the same as those corresponding to the maturation of the number of edges (${\tilde{n}}(m)$ in Table 1). In CS, maturation of the global clustering occurs at the 8-th level, while number of edges maturates at the 9-th level. In the physics dual graph, there is a significant difference between values of local and averaged global clustering: the former is more than two times less than the latter. Most likely, this property is the consequence of the existence of collaborative papers with large degree connected with other papers written by extremely large number of authors. However, such “connecting” authors may have not a close relation of collaboration between each other, and therefore papers authored by them are not necessarily neighbors in the dual graph.

**Table 4:** Maturation indices and maturation values of the graphs’ diameter and characteristic path length.

		All	Math	CS	Phys
	${\bar{n}}$	2831	67	427	2831
*G*	*d*	21	25	26	21
	${\tilde{n}}(d)$	436	3	10	425
	*L*	3.1	7.3	6.1	2.8
	${\tilde{n}}(L)$	–	4	8	–
$G*$	*d*	21	24	26	20
	${\tilde{n}}(d)$	402	4	6	434
	*L*	5.4	8.8	5.2	4.7
	${\tilde{n}}(L)$	329	9	8	430

All notations and definitions are reported in the text. The symbol “–” reflects the fact that the property does not maturate, implying that significant changes in the property’s value occur at all fusion indices, up to the final layer (the reported values are therefore the “asymptotic” ones obtained by fusing all layers).

### Diameter and characteristic path length

The essential measure describing closeness between two particular authors (papers) is the shortest path. Based on this measure two important characteristics of a graph can be calculated. The first is the diameter (*d*)—the maximum shortest path for all pairs of nodes in the LCC. The second is the characteristic path length (*L*)—the mean shortest path for all pairs of nodes in the LCC.

The maturation analysis for *d* and *L* are presented in Table 4. The characteristic path length properties in physics (and, as a consequence, in the whole graph) differ significantly from all other fields: the value of *L* is less than half those in math and CS. However, this value changes significantly on the last layers, therefore, this property is highly dependent on collaborative papers. Interestingly, graphs’ diameters maturate in all fields. The maturation indices in math and CS are close to the values obtained for the number of nodes. Therefore, in these fields papers with relatively large number of authors are basically joint with those who are already in the same community. The difference in physics, instead, indicates that large collaborative papers may influence the network’s community structure.

Similar conclusions can be drawn from the results of the dual graphs, for which even in the case of physics (and the whole network) the characteristics path length maturates. Its maturation appears quite late, but it should be noted that it happens much earlier than edges number maturates. In CS, maturation of both the diameter and the characteristic path length appears earlier than in the primal one. The same is true for the diameter in the field of math. However, characteristic path length in math dual graph maturates later than in the primal graph of this field.

### Centrality and efficiency

As nodes in the networks have very different importance or relevance, various measures of nodes’ centrality have been proposed in the literature. As the distribution of nodes’ centralities in the network (the so-called centrality vector) contains very relevant information on the graphs structure and function, maturation of the centrality vectors is an important signal of the network maturation as a whole. We here report the maturation properties of the mean betweenness and closeness centrality measures, which will be defined momentarily. On the other hand, we also focus here on network’s efficiency, which in real social networks describes the so called “small-world” property—the fact that information transfer is very efficient in such networks^51^.

Node *i*’s betweenness centrality $b_i$ is defined as

8 $$\begin{aligned} b_i = \frac{2}{(N-1)(N-2)} \sum _{j\ne i, k\ne i}\frac{|P(j,k,i)|}{|P(j,k)|}, \end{aligned}$$

where |*P*(*j*, *k*)| is the total number of shortest paths between nodes *j* and *k*, and |*P*(*j*, *k*, *i*)| is the number of shortest paths between *j* and *k* which pass through node *i*. Mean betweenness $\langle b \rangle$ of the graph is obtained by averaging over all nodes, and in the paper we calculate it only for nodes belonging to the LCC.

Node *i*’s closeness centrality $q_i$ is defined as

9 $$\begin{aligned} q_i = \frac{1}{\sum _{j\in V, j\ne i}d(i,j)}, \end{aligned}$$

where *d*(*i*, *j*) is the length of the shortest path between *i* and *j*. Once again, the mean closeness $\langle q \rangle$ is obtained by averaging over all nodes, and limiting ourselves to the set of nodes in the LCC.

Network’s efficiency is defined by

10 $$\begin{aligned} E = \frac{1}{N(N-1)}\sum _{i,j\in V, i\ne j}\frac{1}{d(i,j)}. \end{aligned}$$

Table 5 shows the results for *E*, $\langle q \rangle$ and $\langle b \rangle$. In co-authorship graphs of math and CS papers with extremely large number of authors do not affect the values of the listed properties and, moreover, maturation appears relatively early in both disciplines. This is in agreement with the results of the previous sub-section, where characteristics path length’s maturation was analysed. Moreover, the maturation levels of efficiency and betweenness for these two fields are close to ${\tilde{n}}(L)$. In the dual graphs, the same conclusion can be made only for math and physics. In the case of CS, the dual graph does not instead maturate, and this is the only case in which the dual graph representation seems to provide a less accurate representation of the data. It has to be noticed that, for the CS dual graph and the one for all fields, maturation of centrality and efficiency (not reported here) occurs when $\varepsilon$ is slightly increased (i.e., when $\varepsilon = 0.1$).

**Table 5:** Maturation indices and maturation values of the graphs’ centrality and efficiency indicators.

		All fields	Math	CS	Physics
	${\bar{n}}$	2831	67	427	2831
*G*	*E*	0.40	0.14	0.17	0.43
	${\tilde{n}}(E)$	–	4	7	–
	$\langle q \rangle$	$3.4\cdot 10^{-3}$	$8.8\cdot 10^{-7}$	$7.4\cdot 10^{-7}$	$2.5\cdot 10^{-3}$
	${\tilde{n}}(\langle q \rangle )$	–	6	15	–
	$\langle b \rangle$	$2.4\cdot 10^{-5}$	$4.0\cdot 10^{-5}$	$2.3\cdot 10^{-5}$	$1.9\cdot 10^{-5}$
	${\tilde{n}}(\langle b \rangle )$	–	4	18	–
$G^*$	*E*	0.24	0.13	0.21	0.27
	${\tilde{n}}(E)$	–	9	–	540
	$\langle q \rangle$	$3.2\cdot 10^{-3}$	$4.5\cdot 10^{-4}$	$4.5\cdot 10^{-4}$	$2.4\cdot 10^{-3}$
	${\tilde{n}}(\langle q \rangle )$	–	9	–	444
	$\langle b \rangle$	$4.7\cdot 10^{-4}$	$9\cdot 10^{-4}$	$4.7\cdot 10^{-4}$	$3.8\cdot 10^{-4}$
	${\tilde{n}}(\langle b \rangle )$	–	9	–	447

All notations and definitions are reported in the text. The symbol “–” reflects the fact that the property does not maturate, implying that significant changes in the property’s value occur at all fusion indices, up to the final layer (the reported values are therefore the “asymptotic” ones obtained by fusing all layers).

## Discussion

In summary, we have studied patterns of collaboration in the arXiv database by using the formalism of multilayer higher-order networks, where each layer corresponds to the number of collaborators on publications that are considered for that layer. For layer three, corresponding to three-author publications, and onwards, we have also used higher-order links to connect groups of authors as a much more convenient and theoretically elegant description of group interactions. By doing so, we were able to monitor separately how each relevant topological feature of the network matures toward the value that was measured for the complete classical collaboration network. We have also demonstrated that our representation reveals the true nature of collaborations among researchers, which is fundamentally different when they coauthor a paper in a small group, implying an intense and meaningful research relationship, as opposed to a collaboration in a huge group of coauthors were only very few actually share any noteworthy contact.

In terms of implications for specific research fields, our research shows that different topological features mature at different fusion indices for different research fields. Earlier for fields where the number of authors on a particular publication is traditionally low, as in mathematics, and later for fields where large collaborations are more common, as in physics. Either way, our representation allows us to progressively follow how the final values that determine the topological features of collaboration networks emerge as the fusion index, i.e., the number of layers that have been fused together, increases. This thus offers a completely new and fresh microscopic view into the collaboration patterns of researchers across different disciplines and depth of contact.

It is also worth noting that our research confirms, in line with previous research^20,52^, that the alternative representation of collaboration networks, where hyperlinks connect publications instead of coauthors, yield a better representation in that for these type of collaboration networks all topological features eventually mature as layers are coalesced, whilst in the classical representation some topological feature never mature.

These insights create many possible directions for future research. For example, one viable avenue worth exploring is to customize growth models of hypergraphs that would take into account the fact that a given topological feature must mature at a given stage of fusion. We would thereby obtain a more apt theoretical description of scientific collaboration, which would in turn promise a better understanding of this vital process that upkeeps modern human societies. It would also be interesting to look at the maturation of other network properties, such as the community structure and various centrality measures. Lastly, it would also be worth while exploring how the proposed multilayer higher-order network formalism works in other forms of documented collaboration, such as on patents and legal proceedings. We hope our research will prove inspirational towards this goals in the near future.
